# Radially and Azimuthally Pure Vortex Beams from Phase-Amplitude Metasurfaces

**DOI:** 10.1021/acsphotonics.2c01697

**Published:** 2023-01-04

**Authors:** Michael de Oliveira, Marco Piccardo, Sahand Eslami, Vincenzo Aglieri, Andrea Toma, Antonio Ambrosio

**Affiliations:** † Center for Nano Science and Technology, Fondazione Istituto Italiano di Tecnologia, 20133 Milan, Italy; ‡ Physics Department, Politecnico di Milano, 20133 Milan, Italy; § Fondazione Istituto Italiano di Tecnologia, 16163 Genoa, Italy; ∥ Clean Room Facility, Fondazione Istituto Italiano di Tecnologia, 16163 Genoa, Italy

**Keywords:** vortex beams, Laguerre−Gaussian beams, phase-amplitude metasurfaces, orbital angular momentum

## Abstract

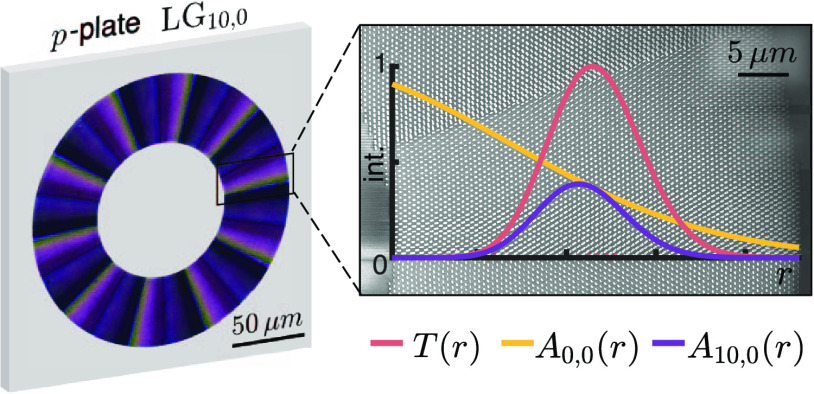

To exploit the full potential of the transverse spatial structure of light using the Laguerre–Gaussian basis, it is necessary to control the azimuthal and radial components of the photons. Vortex phase elements are commonly used to generate these modes of light, offering precise control over the azimuthal index but neglecting the radially dependent amplitude term, which defines their associated corresponding transverse profile. Here, we experimentally demonstrate the generation of high-purity Laguerre–Gaussian beams with a single-step on-axis transformation implemented with a dielectric phase-amplitude metasurface. By vectorially structuring the input beam and projecting it onto an orthogonal polarization basis, we can sculpt any vortex beam in phase and amplitude. We characterize the azimuthal and radial purities of the generated vortex beams, reaching a purity of 98% for a vortex beam with *l* =50 and *p* = 0. Furthermore, we comparatively show that the purity of the generated vortex beams outperforms those generated with other well-established phase-only metasurface approaches. In addition, we highlight the formation of “ghost” orbital angular momentum orders from azimuthal gratings (analogous to ghost orders in ruled gratings), which have not been widely studied to date. Our work brings higher-order vortex beams and their unlimited potential within reach of wide adoption.

## Introduction

The ability to structure light in all its transverse degrees of freedom has led to advances in fundamental science and real-world applications, both classical and quantum.^1^ Particularly significant and utile are vortex light beams, which carry orbital angular momentum (OAM).^2^ Since they are associated with a vortex phase of the form ,^3^ azimuthal phase elements have become ubiquitous for their generation, from spiral phase plates^4^ and computer-generated holograms displayed on spatial light modulators (SLM),^5^ to the use of geometric phase elements such as liquid crystals,^6−8^ or they can be created directly at the source.^9,10^ Recently, a resurgence in vortex generation approaches has emerged with the advent of nanofabrication,^11^ moving away from traditional bulky optical components to subwavelength-structured planar devices that shape the wavefront of light in all its properties, i.e., amplitude, phase, and polarization.

The ease of use and ability to control polarization and phase in metasurfaces have facilitated the realization of many well-established metasurface devices for optical vortex generation, including *q*-plates^12^ and their notable generalization *J*-plates.^13^ However, as with other phase-only devices, they approximate a vortex beam by an incident Gaussian beam modulated by an azimuthal phase profile and subsequently neglect the amplitude term, which defines the annular intensity associated with these modes. As a consequence, the generated beam is not a solution to the paraxial wave equation and thus not an eigenmode of free space. During its propagation, the beam unravels, leading to the excitation of many undesirable radial modes.^14^ An example of a resulting impure vortex mode, with its many concentric rings of intensity, is depicted in Figure S4. This phenomenon has been shown to have a deleterious effect on the detection efficiency in both classical and quantum OAM applications,^15^ the implication of which becomes increasingly prominent for beams carrying larger OAM.

To ensure the invariance associated with eigenmodes during their propagation, the generated vortex beams must be generalized solutions of the paraxial wave equation. Many families of transverse solutions to the paraxial wave equation exist. In cylindrical coordinates, the solutions form a complete, orthonormal basis, called Laguerre–Gaussian (LG) modes of light. These modes require a pair of independent indices to fully describe them: the azimuthal and radial indices,  and *p*. LG beams have garnered much attention because of their circular symmetry, the infinite Hilbert space they provide, and their inherent relation to the quantization of orbital angular momentum (OAM).^16^ They are natural modes of quadratic index media,^17^ making them a viable candidate for free space and optical fiber communication. Their transverse structure is given by^18^
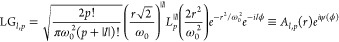
1where ω_0_ is the beam waist,  is the generalized Laguerre polynomial of argument *x*, and *r* and ϕ are the radial and azimuthal coordinates, respectively. The azimuthal component, , is related to a vortex phase profile ψ(ϕ), which results in a “twist” of the wavefront and quantizes the OAM of  per photon.^3^ On the other hand, the radial component, *p*, is related to the transverse amplitude distribution , which results in “ripples” in the beam intensity. Although neglected in the past, radial modes have attracted interest for their diffraction properties as a means to control light propagation in complex media.^19,20^ Recent work has also explored using the radial component as an additional encoding space for quantum^21,22^ and classical information protocols.^23,24^

**Figure 1:**
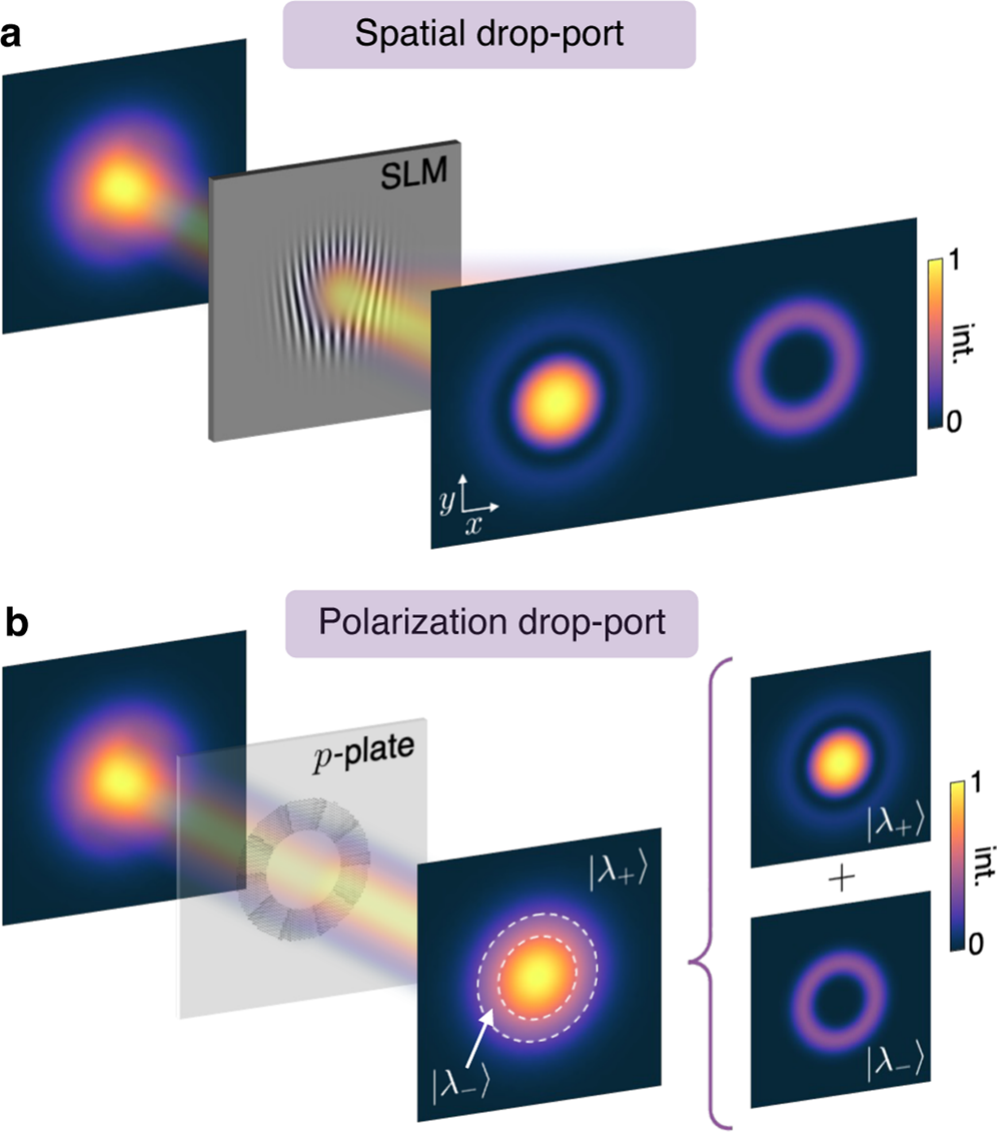
(a) Spatial drop-port, implemented via a grating, is used to carve the amplitude of the incident beam and discard the remainder in a drop-port that can be spatially filtered. (b) Phase-amplitude metasurfaces allow for an alternative approach using polarization as a drop-port. By vectorially structuring the incident beam and projecting it onto an orthogonal polarization state (λ_–_), the desired amplitude can be revealed.

To have control over the azimuthal and radial indices, we need to be able to modulate not only the phase of the incident beam but also its amplitude. One approach is to use an active resonator to facilitate the mode conversion necessary for generating pure OAM modes,^25,26^ but this requires elaborate cavity configurations. Alternative free-space methods have also been demonstrated, which employ complex amplitude modulation using phase-only devices.^27^ This involves carving the amplitude of the incident beam and discarding the remainder in an unwanted drop-port. The common practice is to use a spatial drop-port, as shown in Figure 1a, in which a linear grating diffracts the desired spatial profile, leaving the unwanted amplitude on the main optical path, which can be spatially filtered using an aperture. The drawback of this method is that it requires working off-axis with a diffraction order. Nanostructured silica glass devices have also been proposed to produce pure vortex beams.^28^ However, the devices are restricted to using circularly polarized incident fields, with their practical demonstration being limited to generating vortex beams with low OAM values.^29,30^ On the other hand, vortex beams of high OAM charge and purity have been generated using a two-step process, which combines the high resolution of metasurfaces with complex amplitude modulation achieved using a spatial light modulator to correct for the missing amplitude term.^15^

Here, using polarization as a drop-port, we experimentally demonstrate a generalized method for generating LG modes of high radial and azimuthal purity, which we achieve using a single dielectric metasurface—called the *p*-plate, since it addresses *p*-modes.^31^ The device’s ability to structure light vectorially allows an arbitrary input beam to be modulated in phase and amplitude^32,33^ (after a projection onto the orthogonal polarization state) using a single on-axis transformation, as shown in Figure 1b. We refer to such a combination as a phase-amplitude metasurface.^32^ The compact device also has the advantage that it can be structured as an annular device without sacrificing performance, enabling the fabrication of larger devices while reducing the write time for electron-beam lithography. It follows that the total structured area of the device is inversely proportional to the OAM it imparts (see Figure S5). This results in a reduction in the structured area of up to 75% for a device designed to impart a charge of . Overall, we experimentally show that our device can generate modes with well-defined radial and azimuthal purities, albeit with a simple and practical approach that is readily employable in flat-optics systems, from imaging to quantum applications.

## Results and Discussion

### Ripple-Free Vortex Beams with High Topological Charge

The metasurface device is designed to impart an azimuthal phase, as well as a radially varying polarization transformation that converts part of the incident beam to the orthogonal polarization state. The resulting beam is a vector vortex beam that carries OAM and exhibits a nonuniform polarization distribution. A polarizer, either implemented as a separate element or as a wire-grid grating integrated into the metasurface substrate,^33^ can then be used to select the orthogonal polarization and effectively filter out the excess amplitude and any unconverted light.

**Figure 2:**
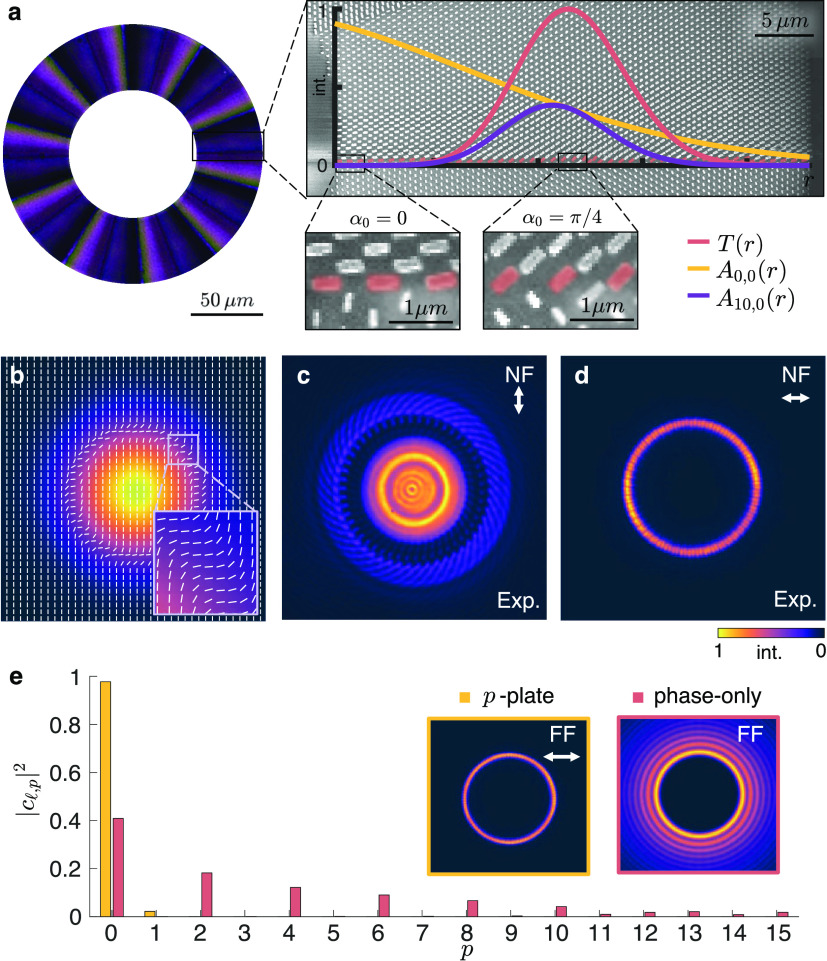
(a) Optical image of a *p*-plate metasurface device designed to generate a pure LG_10,0_ vortex mode. The insets show scanning electron microscope (SEM) images of the device, overlaid with a cross section of the transmission function *T*(*r*) (red), which is required to carve the desired *A*_10,0_(*r*) amplitude (purple) from the incident Gaussian *A*_0,0_(*r*) amplitude (yellow). The transmission function is assimilated into the angles of the pillars α_0_, which vary along the radial direction. Minimum and maximum transmission occur when α_0_ = 0 or π/4, respectively. (b) The simulated vectorial polarization structure of a beam generated directly at the plane of the *p*-plate device designed with  and *p* = 0. The corresponding experimental near-field intensity distributions of the beam projected onto the (c) vertical and (d) horizontal polarization states. (e) The radial *p*-mode spectrum of the vortex beam generated by the *p*-plate compared to the theoretical *p*-mode spectrum of an azimuthal phase-only approach. The insets show the corresponding far-field (FF) intensity distributions.

An example of a fabricated *p*-plate device used to generate a pure LG_10,0_ vortex mode is shown in Figure 2a. The metasurface device consists of many rectangular nanopillars, with the birefringence of each pillar arising from its form factor. Specifically, the desired azimuthal phase profile ψ(ϕ) defines one of the two dimensions of the pillar *L*_*x*_(ϕ), while the other dimension *L*_*y*_(ϕ) is determined by ψ(ϕ) + Δϕ_0_, where Δϕ_0_ is a fixed parameter that corresponds to the phase retardance required to perform the desired polarization conversion to the orthogonal state.^31^ The desired amplitude profile, in this case *A*_10,0_(*r*) (purple line in the inset of Figure 2a), is sculpted from the incident Gaussian amplitude *A*_0,0_(*r*) (yellow line) according to an amplitude transmission function *T*(*r*) (red line). The transmission function is assimilated in the rotation angle of the pillars, α_0_(*r*), to employ a radially varying polarization conversion, which, upon the projection onto an orthogonal state, attenuates the incident amplitude and filters the desired *A*_10,0_(*r*) profile. For example, when the rotation angle of the pillar is α_0_ = π/4, light undergoes a polarization conversion to the orthogonal state that is not attenuated by the polarizer, resulting in the maximum transmission of light at that point. Conversely, when α_0_ = 0, light remains in its original polarized state, which is filtered by the orthogonal polarizer, so no light is transmitted. Therefore, since the desired beam has zero intensity at the center, the metasurface can be preemptively patterned to its characteristic annular shape, as the metasurface in this region would not impart a polarization conversion and light would, in any case, be filtered by the polarizer.

The operating principle of the *p*-plate allows it to be designed to impart the required transformations on any incident wave with known polarization, phase, and amplitude distribution, such as a plane or Gaussian wave. We designed and fabricated the *p*-plates to act on a linearly polarized Gaussian beam, as it is a readily available source in any laboratory. To optimize the generation efficiency of the device, we design the *p*-plates for the specific beam waist ω_s_ of the Gaussian source such that the overlap between the incident intensity profile and that of the desired intensity profile is maximized. The corresponding beam waist ω_0_ of the Gaussian embedded in the target LG mode is then given by . We note that in situations where efficiency can be neglected, this constraint can be lifted by designing the device for an incident plane wave (see Figure S7). In this way, any beam in the laboratory can be easily extended to approximate a plane wave, enabling the *p*-plate device to work with any readily available source, albeit less efficiently since the overlap of intensity profiles will be smaller and more light will be discarded in the drop-port. As a demonstration, we fabricated another *p*-plate device designed to modulate a plane wave, with the corresponding generated mode shown in Figure S7.

To establish the efficacy of the *p*-plate device in generating pure vortex modes, we experimentally demonstrate a *p*-plate that when incident with a Gaussian beam generates an LG_50,0_ vortex beam with a charge of  and no radial modes (*p* = 0). A schematic of the generation and detection experimental setup is shown in Figure S8. The vectorial structure of the beam after the metasurface is shown in Figure 2b. Figure 2c,d shows the corresponding experimental near-field intensity distributions of the generated LG_50,0_ mode when projected onto the vertical and horizontal polarization bases, respectively. The corresponding horizontally polarized component has the characteristic annular intensity distribution of a vortex mode, while the vertically polarized component is the unwanted complementary intensity distribution from which the intensity was carved, leaving behind a dark ring.

To further comment on the purity of the generated vortex beam, i.e., the spread of its energy among different azimuthal () and radial (*p*) modes, the generated vortex beam was decomposed onto the  mode basis. We compute the modal overlap  by simply performing the inner product between the generated vortex beam Ψ(*r*) and the conjugate of the desired mode Φ(*r*) using an SLM and then extract the modal weighting coefficients  as a quantitative measure of purity. A vortex beam has a well-defined radial and azimuthal purity when , i.e., 100% of the power being in the desired LG mode. The inner product is performed experimentally with the details described in the Methods section.

In the case of radial mode purity, the generated vortex was decomposed onto the LG_50,p_ basis, with the measured *p*-mode spectrum shown in Figure 2e. The results confirm a high radial mode purity, with 97% of the generated beam power being in the desired *p* = 0 mode (see Figure S9 for the azimuthal mode spectrum). The Fourier intensity distribution of the vortex beam after it is propagated to the far-field is shown in the inset. We note that no spatial filtering was employed and that the polarization conversion to the orthogonal state allowed any residual light to be filtered by the polarizer. For comparison, the theoretical *p*-mode spectrum is also shown for the case when only an azimuthal phase is used to impart such a high OAM. In this case, the power in the desired *p* = 0 mode drops to 40%, with most of the beam’s energy being spread over higher-order radial modes. Optically, this manifests as concentric rings or radial ripples in the beam’s intensity, as seen in the accompanying inset of Figure 2e.

### Comparing Dielectric Metasurfaces for Vortex Beam Generation

Having demonstrated that *p*-plate metasurfaces can generate vortex beams with high OAM charges and purity, we experimentally compare their performance to those of phase-only metasurfaces. As subjects of the latter, we consider the widely used dielectric metasurfaces, *q*-plates and *J*-plates, as vortex beam generators. The *q*-plate is a spin–orbit coupling device, which uses the geometric phase to convert a circularly polarized incident Gaussian beam to the orthogonal polarization state while imparting an azimuthal phase. These devices are designed using identical pillars, which effectively act as half-waveplates and whose orientation angle varies in azimuth to confer the desired phase profile. On the other hand, the *J*-plate, a significant generalization of the *q*-plate, decouples the dimensions of the pillars, combining both propagation and geometric phase control to imbue any two orthogonal polarization states with independent arbitrary values of OAM,  and . Additionally, we judiciously design the *J*-plates to convert the incident circular polarization to the orthogonal state with opposite handedness. Therefore, in *q*-plates and *J*-plates, the prescribed polarization conversion allows any residual Gaussian beam to be filtered. In contrast to *p*-plate devices, the nanopillars of *q*-plates and *J*-plates do not vary in dimension or orientation angle along the radial direction and as such do not apply any amplitude shaping. Consequently, for *q*-plates and *J*-plates, the beam waist, ω_s_, of the incident Gaussian beam determines the beam waist ω_0_ of the generated LG beam. To ensure a fair comparison, we fabricated all devices using the same design library and methods as before. In addition, the characterization of all devices was performed with the same incident Gaussian beam, so that LG beams carrying the same OAM had the same beam waist, ω_0_.

**Figure 3:**
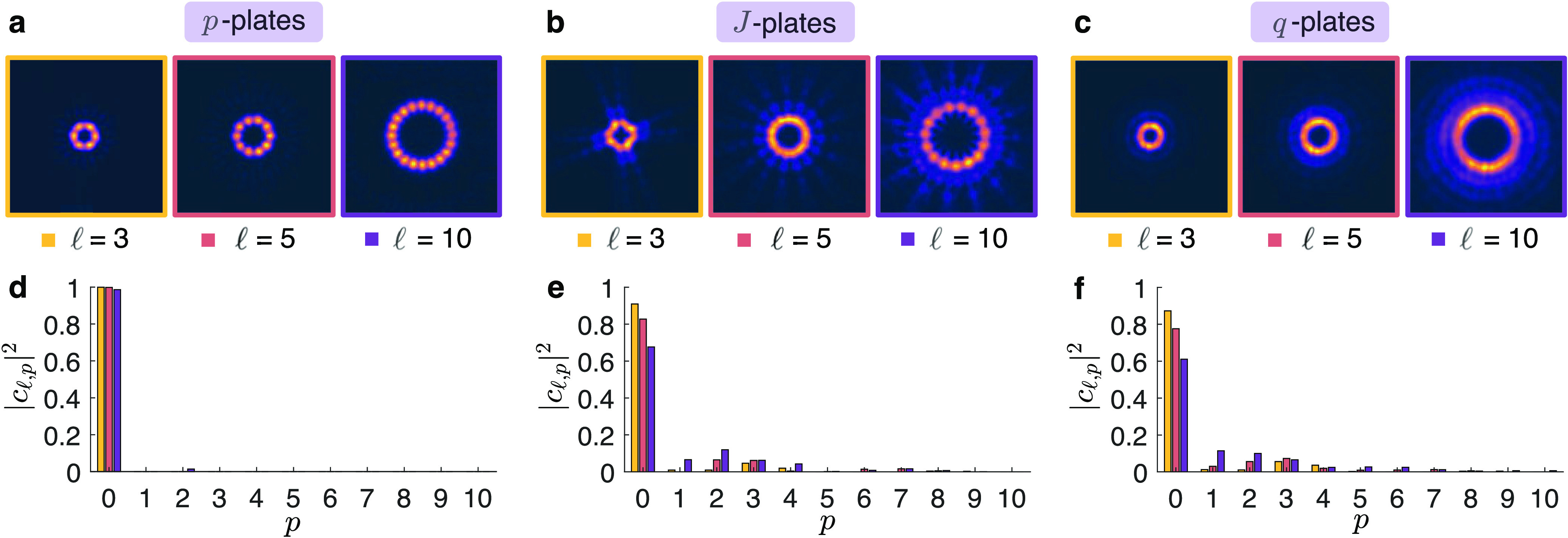
Experimental far-field intensity distributions of vortex beams generated using amplitude and phase control in (a) *p*-plates and phase-only control in (b) *J*-plates and (c) *q*-plates. For each type of device, three devices were designed to impart the topological charge . Corresponding radial *p*-mode spectra for (d) *p*-plates, (e) *J*-plates, and (f) *q*-plates, obtained via a modal decomposition in the  basis. We note that no spatial filtering was used in the generation or detection of the vortex beams.

The comparison between the three types of devices (*p*-plates, *J*-plates, and *q*-plates) used to generate vortex beams of charges  is presented in Figure 3. Visually, many concentric intensity rings can be seen in intensity distribution shown in Figure 3b,c and are more apparent for beams carrying higher OAM. These radial ripples are a consequence of any phase-only approach that modulates a Gaussian beam by an azimuthal phase. In contrast, when the radial degree of freedom is controlled as a means of modulating the amplitude, a single intensity ring appears, as in the case of the *p*-plate shown in Figure 3a. Figure 3d–f shows the corresponding *p*-mode spectra obtained. For *p*-plate devices (see Figure 3d), the power in the desired  mode is greater than 99% for all  modes, indicating high radial mode purity. The *p*-mode spectrum of azimuthal phase-only devices in Figure 3e,f shows how the mode power is spread over a superposition of higher radial modes. This effect becomes more pronounced as the OAM increases, with the power in the desired *p* = 0 radial mode reaching only 61 and 66% for the  mode generated using our *J*-plate and *q*-plate, respectively. These results show a clear advantage for using phase-amplitude modulation for vortex beam generation, particularly for beams with high OAM.

### Ghost Diffraction Orders in Azimuthal Gratings

**Figure 4:**
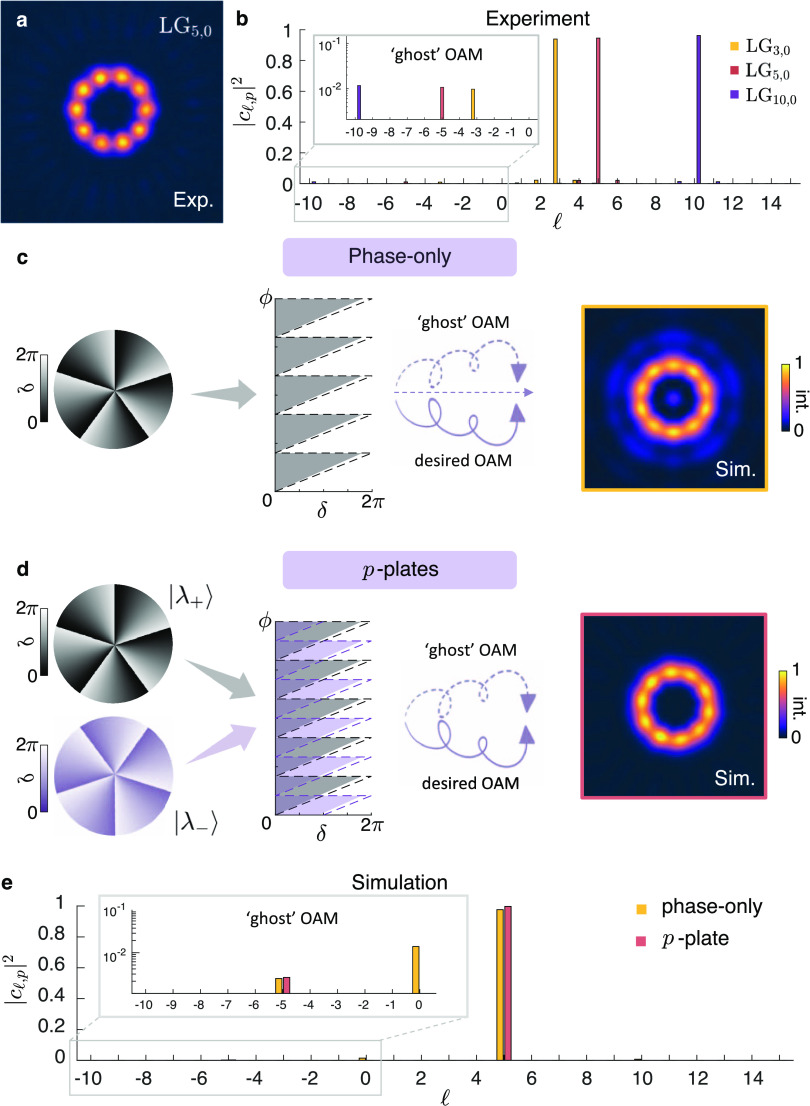
(a) Experimental intensity distribution of a vortex beam generated by a *p*-plate with  and *p* = 0 reveals  azimuthal intensity undulations. (b) The experimental azimuthal -mode spectra for vortex beams generated by *p*-plates with . The inset shows small peaks at contributions of opposite topological charge , which constitute a strong visual effect in panel (a). Illustration of the formation of “ghost” OAM orders as a result of a small deviation in the grating depth, δ, from 2π (dashed line) to 2π M (shaded area) in (c) an azimuthal phase-only grating (which maps to a blazed grating in polar coordinates) and (d) a phase grating with polarization conversion (as in the *p*-plate). In both simulations, each device is designed to impart a topological charge of . (e) The simulated azimuthal -mode spectra for vortex beams generated in panels (c) and (d) showing ghost OAM contributions.

In the experimental intensity images shown in Figure 3, we observe azimuthal undulations in the intensity, reminiscent of a pearl necklace. These “pearls” are an artifact in vortex beams generated by azimuthal phase gratings and have remained unnoticed in the literature, despite being present in previous works.^13,15^ To understand their origin, we consider the case of a *p*-plate whose azimuthal phase is imparted purely by propagation phase in addition to a polarization conversion to the orthogonal polarization state (see the Supporting Information for a discussion on *J*-plate and *q*-plate devices). The intensity distribution for an LG_5,0_ mode generated from a *p*-plate features  pearls, as shown in Figure 4a. Their azimuthal structure alludes to an undesirable contribution of OAM, reminiscent of the intensity distribution of a superposition of interfering vortices of opposite charges,  and , which similarly form well-defined  petal structures. This is confirmed by the azimuthal mode decomposition performed on the vortex beams generated by the *p*-plate devices for , as presented by the corresponding spectra in Figure 4b. Peaks can be seen at the designed azimuthal index, with up to 97% of the power of the generated vortex being in the designed  mode. Additional peaks of the order of a percent are seen at  contributions. Despite being small in contribution, the interference of these modes constitutes a strong visual effect. Indeed, unlike superpositions of radial modes, whose intensity profiles scale with the radial index *p*, superpositions of opposite OAM charges lie on the same radius and have a larger overlap.

The origin of these superfluous OAM orders dates back to the work of Rowland and Michelson^34,35^ on false spectral lines produced by ruled gratings, in which ghost orders were described as originating from periodic errors of grating lines, with small error amplitudes resulting in excessively large ghost intensities. We advance the argument that these ghost orders are not limited to linear gratings but are susceptible to any diffraction grating elements, including azimuthal gratings. In the case of the latter, the relation becomes apparent when the azimuthal profile is mapped to polar coordinates, revealing a linear blazed grating (see Figure 4c). It follows then that a small deviation in the grating depth, δ, which in the case of metasurfaces may result from fabrication errors that deviate from the designed pillar size or height, will lead to a cross-coupling of adjunct ghost OAM modes.

To validate their presence, we performed numerical simulations, in which we show that a small deviation (on the order of 10%) in the azimuthal grating depth produces the so-called ghost OAM orders. We consider two cases for generating an  vortex beam. The first applies a phase-only azimuthal phase (see Figure 4c), which unwraps to a blazed grating with  phase jumps in polar coordinates. The second applies an azimuthal phase as well as an arbitrary polarization conversion, as in the case of *p*-plates (see Figure 4d). In this case, the form birefringence of the metasurface structure allows each orthogonal polarization state to be addressed with an arbitrary phase profile. For *p*-plates, the azimuthal phase profile seen by the orthogonal polarization states is the same. This results in an unwrapped blazed grating with  phase jumps. The presence of ghost OAM orders, in both cases, is confirmed by the simulated azimuthal modal spectrum, shown in Figure 4e. For the phase-only approach, ghost OAM orders appear at multiples of the designed charge , whereas for the *p*-plate device, there is only one appreciable OAM diffraction peak at . The polarization conversion in the *p*-plate device results in a cross-coupling of ghost orders that suppresses their effect, with the OAM orders being spaced by  (see the Supporting Information). In both cases, the contribution at  manifests as the  pearls in the intensity profile of the vortex beam. Consequently, the numerical result for the *p*-plate device agrees with the experimental results, in that a small deviation in the grating depth manifests as small contributions of ghost OAM orders, with the first OAM diffraction order at . Nonetheless, we emphasize that although this is a visually pronounced effect, it corresponds to a very small contribution of around a few percent. The azimuthal mode purity of the generated beams is above 97%, which is comparable to the radial purity results for the same devices.

## Conclusions

We have demonstrated the generation of high-purity vortex beams from a single metasurface, with complete control of both azimuthal and radial components. The ability to structure light vectorially and, therefore, to use polarization as a drop-port provides a compact on-axis conversion system that is easy to implement in practical flat-optics systems. Furthermore, the subwavelength resolution of the device allows for the structuring of pure vortex beams of very high OAM, highlighted in the demonstration of a vortex beam with an  charge. Our results show the benefit of using *p*-plate devices for radial mode purity, which outperform other well-established vortex-generating metasurfaces (*J*-plates and *q*-plates). As such, *p*-plates could be of interest in any vortex application in which the radial mode purity cannot be neglected.

The generalized approach is not limited to pure vortex modes but rather allows us to harness the full resource of LG modes with any desired  and *p*-mode distributions. In fact, the ability to perform phase and amplitude modulation allows us to sculpt many other families of paraxial beams. Exploiting the control over the complete transverse spatial degree of freedom is significant for the emerging field of high-dimensional quantum information, boosting communication channels with higher encoding capacities^24^ and increasing noise robustness in entanglement distributions.^36^ We foresee these devices as a very convenient and powerful approach, which could further drive the uptake of higher-order vortex modes.

## Methods

### Metasurface Fabrication

The dielectric metasurface devices consist of amorphous silicon nanopillars on a fused silica substrate, arranged in a hexagonal closed-packed lattice. The metasurfaces are designed to operate in the near-infrared at 1064 nm. A phase library of nanopillars was simulated using a finite-difference time-domain software (Lumerical), together with a complex-value refractive index measured by ellipsometry. The height of each pillar was fixed at 600 nm, while the dimensions and orientation angle of the pillars were allowed to vary to impart a different phase delay between the *x* and *y* components of the field (by exploiting form birefringence). This allowed the required transformations to be implemented using either propagation or geometric phase. The fabricated devices are 200 μm in diameter and where applicable are designed to accept a focused Gaussian with a beam waist of 92 μm. Particularly, the *p*-plate devices were preemptively structured as annular rings by defining the annular region as the region where the intensity transmission function of the device is above 5%.

The fabrication of the metasurfaces proceeded as follows: an a-Si film of 600 nm was deposited onto the fused SiO_2_ substrate by plasma-enhanced chemical vapor deposition (STS LPX PECVD system). To ensure no charging effects during electron-beam exposure, a 10 nm thick Au film was deposited on top of the poly(methyl methacrylate) layer by physical vapor deposition. The exposure was carried out using a Raith150 Two electron-beam lithography system, with a beam energy of 20 keV and a beam current of 140 pA. A thin layer of Cr was deposited on the sample and then subjected to a lift-off process to reveal a hard mask of the metasurface design. This mask was transferred to the a-Si film using a plasma-enhanced reactive ion etching system (Sentech SI500), which was optimized for etch depth and sidewall verticality.

### Measuring the Purity of Vortex Beams

The azimuthal and radial purities of the generated vortex beams are measured using a well-established phase flattening approach, called modal decomposition,^37^ with the idea of unraveling the wavefront into a Gaussian-like mode. To do this, the generated beam is imaged via a 4f telescope onto a phase-only SLM (HOLOEYE GAEA-2), on which we display complex amplitude holograms of the conjugate modes in our LG basis, as outlined in ref (37). We use the beam waist ω_0_ of the embedded Gaussian to define the hologram used in the complex amplitude modulation. We operate the SLM at the first diffraction order in the reflection mode, although Figure S8 shows it in transmission mode for simplicity. We compute the optical overlap by means of a lens and measuring the Fourier-plane on-axis pixel intensity using a camera. A pinhole is placed before the camera (Spiricon LT665) to block unwanted diffraction orders. In this way, we extract the weighting coefficients  that describe the contribution of each of the modes in our basis. We employed background subtraction to remove noise from the acquired intensity images. We note that a combination of quarter- and half-waveplates (not shown in Figure S8) were used to ensure the correct polarization conversion in the generation and detection steps.

## Supporting Information Available

Discussion on the generation of ghost OAM orders by *J*-plates and *q*-plates, *p*-plate conversion efficiency, reduction in the writing area of the p-plate device as a function of the OAM, schematic of the experimental generation and detection setup, and optical images of LG_50,0_*p*-plate devices for both plane wave and Gaussian incident waves (PDF)
