# Supplementary material for: Radially and Azimuthally Pure Vortex Beams from Phase-Amplitude Metasurfaces
Source: ACS Photonics. 2023 Jan 4;10(1):290–7. doi: 10.1021/acsphotonics.2c01697 (PMC9853941; doi:10.1021/acsphotonics.2c01697)
Supplement: Supplementary file 1 — ph2c01697_si_001.pdf [file ph2c01697_si_001.pdf]

# Supplementary Material to: Radially and azimuthally pure vortex beams from phase-amplitude metasurfaces

Michael de Oliveira,[†,‡,∥] Marco Piccardo,[†,∥] Sahand Eslami,[¶] Vincenzo Aglieri,[§] Andrea Toma,[§] and Antonio Ambrosio[*,†]

†*Center for Nano Science and Technology, Fondazione Istituto Italiano di Tecnologia, 20133 Milan, Italy*

‡*Physics Department, Politecnico di Milano, 20133 Milan, Italy*

¶*Fondazione Istituto Italiano di Tecnologia, 16163 Genoa, Italy*

§*Clean Room Facility, Fondazione Istituto Italiano di Tecnologia, 16163 Genoa, Italy*

∥*Contributed equally to this work*

E-mail: antonio.ambrosio@iit.it

## ‘Ghost’ OAM orders generated by $J$-plates and $q$-plates

In Figure 4a of the main text, we presented and discussed the azimuthal $\ell$-mode purity of the vortex modes generated by the $p$-plate devices. Here, we extend those results to include the azimuthal $\ell$-mode decomposition results for the phase-only metasurface devices, namely the $J$-plates and $q$-plates, shown in Figure S1. Recall that the form birefringence of metasurfaces allows to impart two different phase profiles, i.e., $\ell_1, \ell_2$ on two orthogonal polarization states, $|\lambda_+\rangle$ and $|\lambda_-\rangle$, respectively. This allowed us to design and fabricate only two $J$-plate devices that act on circularly polarized light: the first with $\ell_1 = 1, \ell_2 = 3$ and the second with $\ell_1 = 5, \ell_2 = 10$. On the other hand, the $q$-plate design is restricted to only impart conjugate OAM, $\ell_1 = -\ell_2$, to orthogonal circular polarization states, such that three $q$-plate devices were fabricated, one for each charge $\ell = 3, 5, 10$. In the case of $J$-plates, the decomposition reveals peaks at the desired charges $\ell = 3, 5, 10$ respectively, with ‘ghost’ OAM contributions of a few percent at the conjugate of the designed charge. For $q$-plates nearly all power is in the desired $\ell$ mode, with no auxiliary OAM contributions. To understand the origin of these OAM diffraction orders, or the lack there of, we expound the description in the main text to include the effect of a change in phase depth in azimuthal gratings for the case of $J$-plates and $q$-plates.

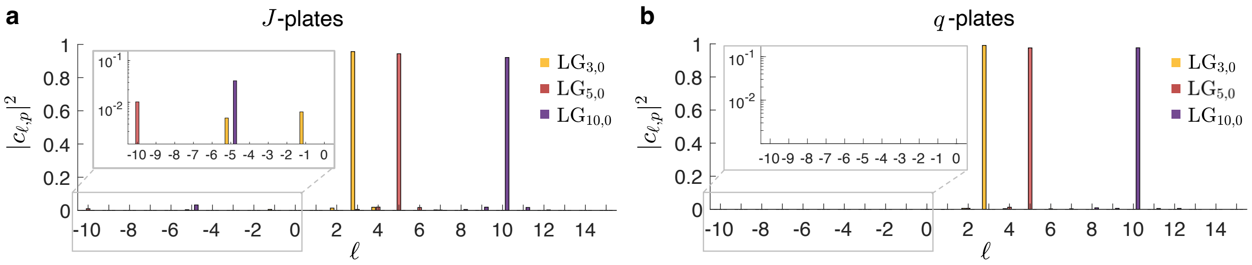


Figure S1: Corresponding azimuthal $\ell$-mode spectra of vortex beams generated using phase-only control in (a) $J$-plates and (b) $q$-plates, obtained via a modal decomposition in the $LG_{\ell,p}$ basis. For each type of device, three devices were designed to impart the topological charge $\ell = 3, 5, 10$. We note that no spatial filtering was used in the generation or detection of the vortex beams.

Recall that the $p$-plate devices were designed such that $\ell_1 = \ell_2 = \ell$. Unwrapping the

required azimuthal phase profile reveals a blazed grating with $\ell$ phase jumps for each orthogonal polarization, as seen in Figure S2a, with a total of $2\ell$ phase jumps. These desired azimuthal phase delays are imparted via a propagation phase that is related to the dimensions of the nano pillar. This manifest visually as $2\ell$ wedge sectors in the device design. We note that the rotation angle for the nano pillars is kept constant in the azimuthal direction and only varies in the radial direction, so as to structure the beam vectorially and sculpt the desired amplitude profile. Since the imparted phase depends on the dimensions of the pillars, any small deviation from the designed pillar size will affect the grating depth. As discussed in the main text, such a small deviation in the grating depth leads to a cross-coupling of unwanted 'ghost' OAM modes, with the first OAM diffraction order being at $-\ell$. More specifically, the 'ghost' orders are formed at multiples of $|\ell_1| + |\ell_2| = 2|\ell|$, centered about the desired charge.

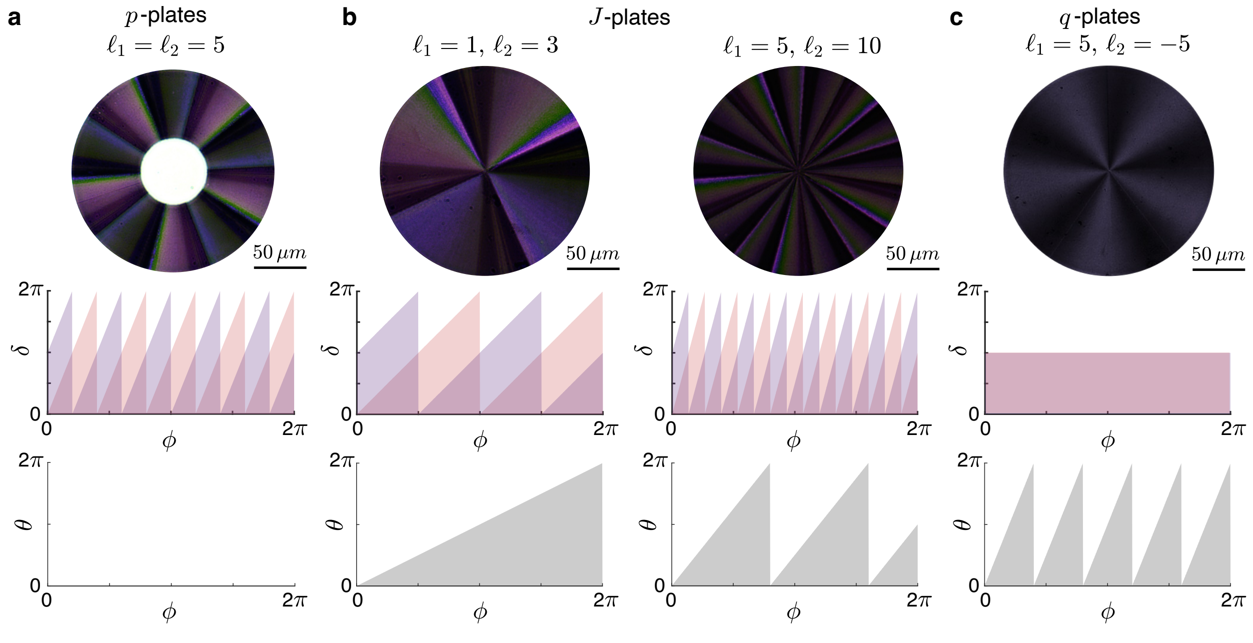


Figure S2: Optical images of the fabricated metasurfaces for (a) $p$-plate, (b) $J$-plates and (c) $q$-plates, with the OAM charge $\ell_1$, $\ell_2$ imparted on each orthogonal polarization state, respectively. Th middle row shows the unwrapped phase delays $\delta$ for both designed orthogonal polarization states as a function of azimuthal angle $\phi$. The bottom row shows the rotation angle $\theta$ of the nano pillar for a fixed radial coordinate as a function of azimuthal angle $\phi$.

Next, we extend this to the generalized case of the $J$-plates, which combine both prop-

agation phase and geometric phase control to impart orthogonal circular polarization states with different amounts of OAM, $\ell_1$ and $\ell_2$, while also performing a polarization conversion. The required phase delays are given by $\delta_+ = 1/2(\ell_1+\ell_2)\phi$ and $\delta_- = 1/2(\ell_1+\ell_2)\phi-\pi$, and the rotation angle of the pillars is given by $\theta = 1/4(\ell_1-\ell_2)\phi$.[1] Figure S2b shows the required unwrapped phase delays, in which one can count $|\ell_1|+|\ell_2|$ phase jumps, and the corresponding rotation angles, which vary along the azimuthal coordinate, for the two fabricated $J$-plate devices. Following the same reasoning as for the $p$-plates, 'ghost' OAM diffraction orders appear at multiples of $|\ell_1| + |\ell_2|$, centered about the desired charge. This is indeed the case in Figure S1, with the first order contributions at the conjugate of the desired charge. An exemplary case is that of the desired charge of $\ell_2 = 3$, where we see a first order peak at $\ell = -1$ and a second order peak at $\ell = -5$ which are equally spaced by $|\ell_1|+|\ell_2| = 1+3 = 4$ modes. Similarly, for the device with $\ell_1 = 5$ ($\ell_2 = 10$), we see a first order peak at the conjugate of the orthogonal charge, $-\ell_2 = -10$ ($-\ell_1 = -5$).

Lastly, we turn our attention to the azimuthal decomposition of the $q$-plate devices, in which we see no 'ghost' OAM orders. In contrast to the $p$-plate and $J$-plate devices, the $q$-plate device imparts the desired azimuthal phase using only geometric phase. Essentially, the device is designed using identical pillars, whose orientation angle varies in azimuth to impart the conjugate azimuthal phases $\ell_1 = -\ell_2$ to orthogonal circular polarization states. The corresponding phase delay and rotation angles are shown in Figure S2c. As opposed to the case of $p$-plates and $J$-plates, fabrication errors in the size of the pillars affect the efficiency of the polarisation conversion and not the azimuthal grating depth, as the latter is geometrically defined by the rotation angles of the pillars. As a result, the generation of 'ghost' OAM orders is less susceptible in geometric phase elements, although the effect is still possible if the applied azimuthal phase grating is changed in some other way. Nevertheless, we reiterate that $q$-plates are not suitable in the context of this paper, as they do not structure the beam amplitude (they are phase-only devices) and thus generate vortex modes that unravel during propagation into a superposition with many radial modes.

## $p$-Plate conversion efficiency

When applying a single-step amplitude shaping (regardless of the device used), the power conversion efficiency depends on the radial distribution of both the source and target $\mathrm{LG}_{\ell,p}$ mode. It is then important to consider the incident wave, in which case we choose a Gaussian beam as it is a readily available source in the laboratory. We can calculate the maximum modal power content $\eta$ of the generated beam by taking the overlap between the Gaussian beam incident on the metasurface (or other device that implements amplitude shaping, such as an SLM) and that of the target $\mathrm{LG}_{\ell,0}$ mode. This is given by[2]

$$\eta = e^{|\ell|}\left[\frac{\omega_s}{\omega_0}\sqrt{\frac{|\ell|}{\left(\frac{\omega_s}{\omega_0}\right)^2-1}}\right]^{-2|\ell|}\Gamma\left(\frac{1}{2}+|\ell|\right)\frac{\omega_0}{\sqrt{\pi}\omega_s}, \tag{1}$$

where $\omega_s$ is the beam waist of the Gaussian source, $\omega_0$ is the beam waist of the embedded Gaussian in the $\mathrm{LG}_{\ell,0}$ mode and $\Gamma(...)$ is the gamma function. There is an optimum choice for the beam waist $\omega_s$ of the Gaussian source that maximizes the overlap with the mode, which occurs when $\omega_s/\omega_0 = \sqrt{|\ell|+1}$.This sets an upper bound in conversion efficiency that is independent of the technique used to apply the amplitude and phase modulation. The maximum modal power achievable as a function of the $\ell$ index is shown in Figure S3 (bars), reaching a maximum of 29% for $\ell = 3$, 23% for $\ell = 5$ and 16% for $\ell = 10$. We see that a higher $\ell$ index results in lower conversion efficiency, as there is less overlap with the incident Gaussian beam and more light is discarded. The measured power conversion efficiency for each of the fabricated $p$-plate devices, are shown in Figure S3 (black dots). They were calculated as the fraction of power of the beam incident on the metasurface that is converted into the target $\mathrm{LG}_{\ell,0}$ mode, as measured after the polarizer. These values are consistent with the theoretical maximum bounded by the technique itself, with experimentally measured efficiencies of 25% for $\ell = 3$, 19% for $\ell = 5$ and 13% for $\ell = 10$. It shows a clear benefit of using $p$-plates for the generation of pure vortex beams with large OAM charge and well

define radial index, as the efficiency remains sizeable even for very large $\ell$ values.

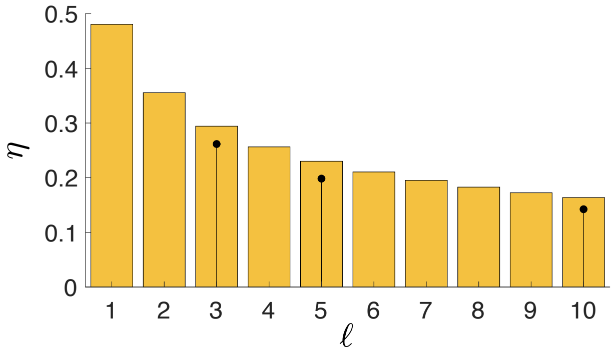


Figure S3: Theoretical maximum (yellow bars) and experimentally measured (black dots) power conversion efficiencies $\eta$ for $p$-plate devices of charge $\ell$. More specifically, $\eta$ is the fraction of the input beam power that is contained in the target $\mathrm{LG}_{\ell,0}$ mode.

## Supplementary figures

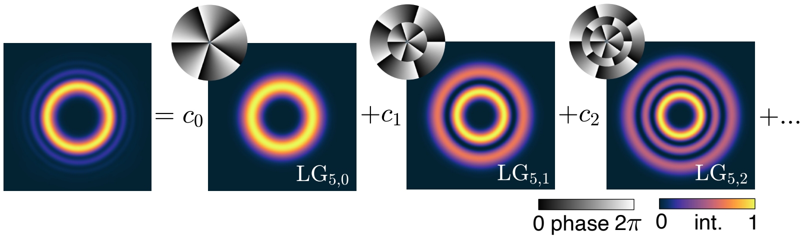


Figure S4: A simulation of a Gaussian beam modulated by an azimuthal phase aperture produces a vortex mode with many concentric intensity rings. During its propagation, the power of the desired azimuthal mode is spread over many higher-order radial modes.

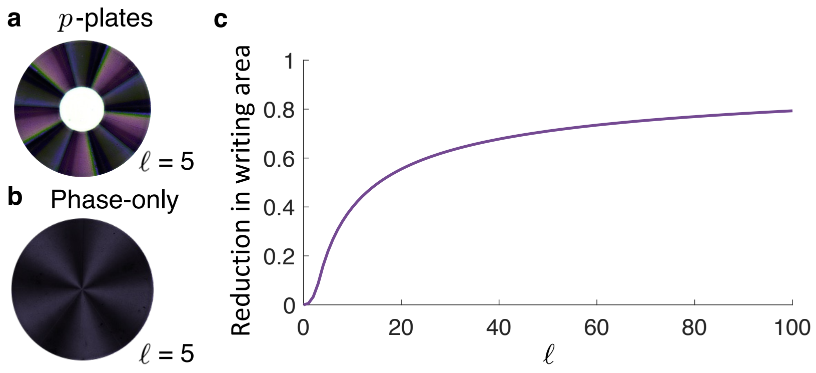


Figure S5: Optical image of the fabricated (a) $p$-plate and (b) phase-only metasurfaces. (c) Simulation showing the reduction in the writing area of the $p$-plate device as a function of the OAM it imparts as compared to that of a phase-only metasurface. This is calculated as the complement of the area of the $p$-plate to that of the phase-only device. The active area of the $p$-plate is selected where the intensity transmission efficiency is above 5%. This results in a characteristic ring shape of $p$-plate devices and allows to fabricate larger devices with higher OAM, while reducing the electron beam lithography writing time.

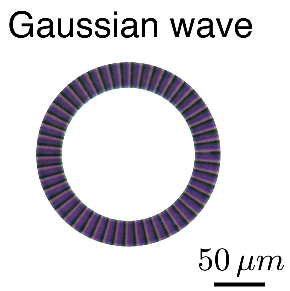


Figure S6: Optical image of the fabricated $p$-plate device with $\ell = 50$ and $p = 0$, designed to modulate an incident Gaussian beam.

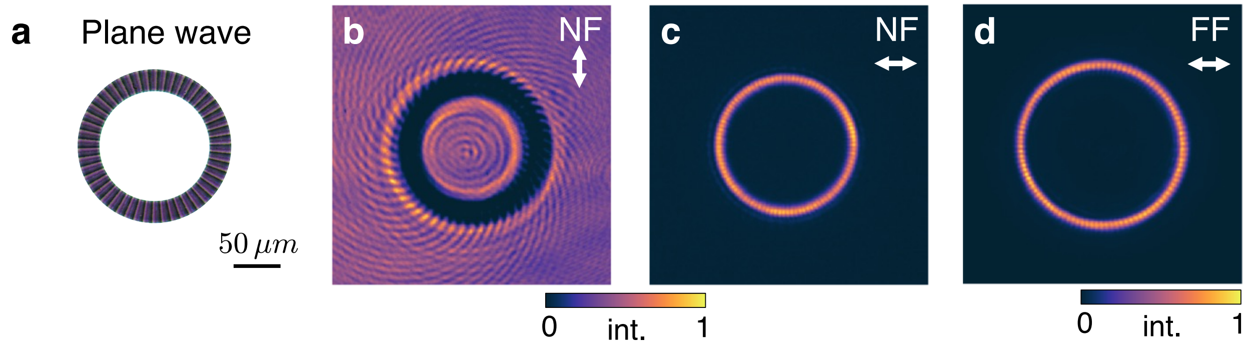


Figure S7: (a) Optical image of the fabricated $p$-plate device with $\ell = 50$ and $p = 0$, designed to modulate an incident plane wave. (b-c) The experimental near-field (NF) intensity distributions of the generated beam from the plane wave $p$-plate device shown in (a). (b) The vertically polarized component is the unwanted complementary intensity distribution from which the intensity was carved, while (c) the horizontally polarized component has the characteristic annular intensity distribution of a vortex mode. (d) The corresponding far-field (FF) intensity distribution of vortex beam in (c).

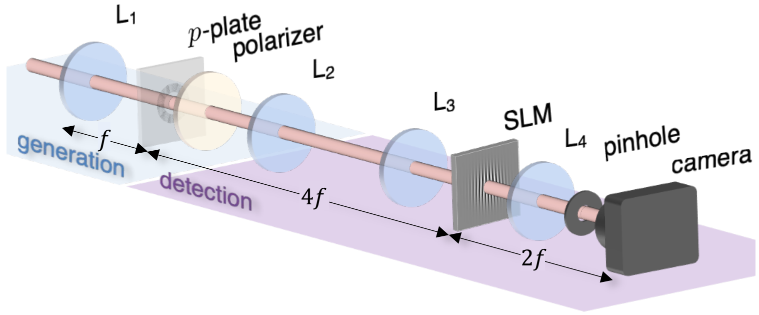


Figure S8: Schematic of the generation and detection of vortex beams from a $p$-plate metasurface. A focused, linearly polarized Gaussian beam propagates through the $p$-plate and exits the polarizer as a pure vortex beam. The purity of the vortex mode is characterized by performing optical overlap measurements. In the near-field (NF) of the beam, complex-amplitude holograms are displayed on a spatial light modulator (SLM) and the resulting on-axis intensity in the Fourier plane is measured using a camera. The SLM operates in reflection but for simplicity is shown in transmission.

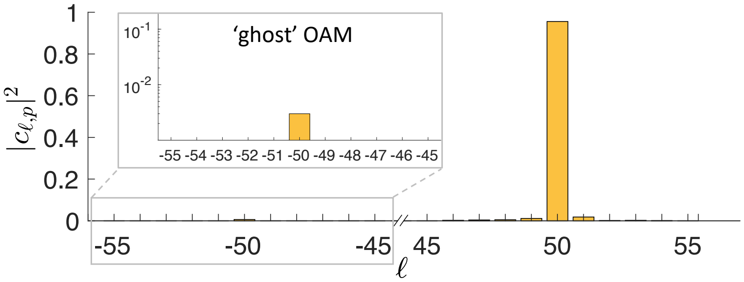


Figure S9: The azimuthal $\ell$-mode spectrum of the vortex beam generated by a $p$-plate with $\ell = 50$ and $p = 0$. The peak power contribution is at the designed charge. The inset shows a small contribution of a 'ghost' OAM order at the opposite charge $-\ell$. This small contribution results in the azimuthal intensity undulations, so called 'pearls', in far-field intensity image shown in the inset of Figure 2d.

# References

(1) Devlin, R. C.; Ambrosio, A.; Rubin, N. A.; Mueller, J. B.; Capasso, F. Arbitrary spin-to–orbital angular momentum conversion of light. *Science* **2017**, *358*, 896–901.

(2) Piccardo, M.; Ambrosio, A. Arbitrary polarization conversion for pure vortex generation with a single metasurface. *Nanophotonics* **2021**, *10*, 727–732.
